# Decision-making for intensive rehabilitation in patients with Trousseau syndrome: Insights from a case series

**DOI:** 10.1097/MD.0000000000034097

**Published:** 2023-06-30

**Authors:** Shinichi Takeshima, Nobuyuki Kawate

**Affiliations:** a Department of Rehabilitation Medicine, Showa University School of Medicine, Yokohama, Kanagawa, Japan.

**Keywords:** intensive rehabilitation, modified Rankin scale, the convalescent rehabilitation ward, the functional independence measure, Trousseau syndrome

## Abstract

Patients with Trousseau syndrome, a common complication of advanced cancer, typically have poor survival. For that reason, there is a need to determine the effectiveness of rehabilitation treatment and develop a comprehensive treatment strategy earlier than that in the general stroke population. We investigated the relationship between physical function and its outcome 1 month after the start of intensive rehabilitation treatment in patients with Trousseau syndrome, to obtain insights for determining the indications for intensive rehabilitation in these patients.

**Patient concerns:**

The development of Trousseau syndrome may worsen the performance status (PS), often necessitating a reevaluation of the indications for treatment of the primary cancer. Furthermore, the primary cancer may progress during rehabilitation therapy.

**Diagnoses:**

These patients were diagnosed with Trousseau syndrome.

**Interventions:**

All patients underwent training under the supervision of a therapist for 2 to 3 hours per day, 7 days per week, with a focus on exercise therapy. The functional independence measure (FIM) 1 month after admission to the convalescent rehabilitation ward, modified Rankin scale (mRS) score on admission and on the date of last assessment, and its outcome was examined.

**Outcomes:**

The time from stroke onset to admission to rehabilitation ranged from 22 to 60 days. Primary cancers were lung, bladder, prostate, ovarian, uterine, and unknown primary. Four patients had advanced cancer with distant metastasis. Two patients were discharged to home with independent activities of daily living (ADL) status. Two patients were transferred to palliative care, and 3 patients died. The 2 patients with independent ADL status had a mean motor score of 90 and a mean cognitive score of 30 on FIM, while the other 5 patients had a mean motor score of 29 and a mean cognitive score of 21 at 1 month of admission. Patients with mRS > 3 on admission did not have independent ADL status at 1 month.

**Lessons:**

Intensive rehabilitation therapy may be indicated for patients with Trousseau syndrome who are expected to improve physical function after approximately 1 month of rehabilitation. Palliative care should be considered if recovery is inadequate.

## 1. Introduction

Patients with cancer are at high risk of cardiovascular events.^[1]^ Within the first 6 months of cancer diagnosis, the risk of stroke increases substantially in correlation with the stage of cancer.^[2]^ Thus, highly active cancer is an established risk factor for ischemic stroke.^[3]^ Trousseau syndrome refers to stroke symptoms caused by hypercoagulability induced by malignancy.^[4]^ Trousseau syndrome is a common complication of adenocarcinomas such as lung, pancreatic, gastric, ovarian, and prostate cancers.^[4–7]^ Adenocarcinoma cell-derived mucin is believed to react with L-secretin in leukocytes and P-secretin in platelets, leading to platelet aggregation without thrombin production.^[8]^ Although anticoagulation therapy is often used clinically, treatment for the primary cancer is the most effective treatment for prevention of recurrence.^[9]^ However, since cancer-associated infarction tends to be severe,^[10]^ the development of Trousseau syndrome may worsen the performance status (PS), often necessitating a reevaluation of the indications for treatment of the primary cancer. Exercise training can improve the functional capacity of stroke patients and reduce the subsequent risk of cardiovascular events.^[11]^ Therefore, intensive rehabilitation treatment can help improve the PS of these patients, thereby restoring the indications for cancer treatment. However, treatment of primary cancer is rarely continued during rehabilitation treatment, and the cancer itself may progress during this period. In addition, patients with advanced cancer have a limited survival time, and prolonged hospitalization due to treatment means a shorter period of time spent at home. Therefore, in Trousseau syndrome, it is important to determine the effectiveness of rehabilitation treatment and to develop a comprehensive treatment strategy earlier than that in the general stroke population.^[12]^ Despite this, very few reports have discussed this point. In this retrospective case-series study, we assessed the relationship between physical function and outcomes 1 month after the start of intensive rehabilitation treatment in patients with Trousseau syndrome who had completed treatment for the acute phase. Our findings may provide insights for determining the indications for intensive rehabilitation treatment.

## 2. Materials and methods

This was a retrospective case-series study. Between January 2017 and December 2020, a total of 636 patients who were admitted to the Rehabilitation Department of our hospital after acute stroke treatment were evaluated. Of these, cases that qualified the following criteria were defined as having Trousseau syndrome:

1. Patients who were undergoing cancer treatment at the time of stroke onset or who were newly diagnosed with cancer at the time of stroke.
2. Patients classified as having a stroke of undetermined etiology according to the Trial of Org 10172 in Acute Stroke Treatment classification,^[13]^ and in whom the cause of cerebral infarction other than malignancy could not be identified.

Cases without malignant tumor complications, cases with a history or complications of malignancy but not under treatment, cases clearly affected by risk factors such as hypertension, diabetes mellitus, and dyslipidemia, and cases due to other obvious causes were excluded because they were considered to have little association with malignancy.

The functional independence measure (FIM) 1 month after admission and the modified Rankin scale (mRS) score on admission and at the most recent assessment were investigated. Furthermore, we considered the tendencies of FIM and mRS scores in patients who achieved independent daily living and those who did not. The FIM is a method for evaluation of activities of daily living (ADL) status consisting of 13 motor items (13–91 points) and 5 cognitive items (5–35 points), with higher scores indicating greater independence. The mRS is used to evaluate the degree of independence in life after stroke onset, classified into 7 levels from grade 0 (asymptomatic) to grade 6 (dead).

All patients underwent training, mainly exercise therapy, under the supervision of a therapist for 2 to 3 hours daily, in a dedicated training room. The rehabilitation protocol was individualized through close information exchange among the staff. This study was approved by the Showa University Ethics Committee (approval number 21-109-B). For this report, we provided a sufficient explanation to the participants in question and obtained written consent from them.

## 3. Results

### 3.1. Case background

Seven patients (3 male and 4 females; age range, 50–88 years) were included. The time from stroke onset to admission to our hospital ranged from 22 to 60 days. The hospitalization period ranged from 19 to 161 days. Four patients had hypertension, 1 had diabetes mellitus, and 1 had atrial fibrillation, all of whom were receiving oral medication. Deep vein thrombosis was detected in 5 cases. The details about primary cancer, clinical stage, and histopathology are showed in Table 1.

**Table 1:** Clinicopathological characteristics of the study population.

Case	Age	Sex	Onset to admission (d)	Hospitalization period (d)	Primary cancer	Tissue diagnosis	Clinical stage	Stroke recurrence
1	65	M	56	101	Bladder	Undifferentiated ca.	IV	+
2	85	F	44	28	Undetermined	Undetermined	Unclear	+
3	77	M	22	45	Lung	Adenocarcinoma	III	−
4	75	F	60	161	Ovarian	Clear cell ca.	IC	−
5	88	M	22	23	Prostate	Undetermined	IV	+
6	51	F	36	25	Ovarian	Endometrioid adenocarcinoma	IV	−
7	50	F	34	19	Uterine	Endometrioid adenocarcinoma	IV	+

Ca = carcinoma, F = female, M = male.

### 3.2. FIM after 1 month and mRS trends and outcomes

Two patients in this series regained the ability to independently perform the daily activities and were discharged. Two patients were transferred to palliative care, and 3 patients died during the observation period. Among them, case 1 was able to walk with light assistance approximately 2 months after admission (4 months after stroke onset) but died of cancerous pleurisy 128 days after admission.

One month after admission, the 2 patients who were independent in daily activities averaged 90 points on motor items of FIM and 30 points on cognitive items of FIM, while the other 5 patients averaged 29 points on motor items of FIM and 21 points on cognitive items of FIM (Table 2). Judging from the mRS scores, patients who required assistance with walking activities upon admission did not achieve independence in ADL.

**Table 2:** Outcomes and physical function of patients with Trousseau syndrome.

Case	Outcome	FIM (1 mo after admission)		mRS score at admission	mRS score at last confirmation date
		Motor items	Cognitive items		
1	Death	39	34	5	6
2	Death	18	14	4	6
3	Death	18	13	5	6
4	Palliative care	37	17	5	4
5	Palliative care	33	27	4	4
6	Home	90	30	3	2
7	Home	90	35	2	2

FIM = functional independent measure, mRS = modified Rankin scale.

### 3.3. Clinical manifestations

Case 1 had symptoms of mild impairment of consciousness, left hemispatial neglect and memory impairment, and severe left hemiplegia. Case 2 showed left hemispatial neglect, mild attention impairment, and mild left hemiplegia. Case 3 showed nonfluent aphasia, dysarthria, and mild left hemiplegia. Case 4 showed mild impairment of consciousness, left hemispatial neglect, and mild bilateral hemiplegia including symptoms of previous stroke. Case 5 showed mild left hemiplegia and dysarthria. Case 6 had nonfluent aphasia and dysarthria. Case 7 had nonfluent aphasia and right ataxia.

### 3.4. Treatment strategy for primary cancer

Three patients with gynecological cancer underwent tumorectomy after the first stroke (21–52 days) for the purpose of tumor reduction or staging. One patient (case 4) received palliative care because she did not wish to undergo further treatment, while chemotherapy was started after rehabilitation treatment in the other 2 patients (cases 6 and 7). No aggressive treatment was performed in cases of lung cancer, prostate cancer, and cancer of unknown primary (cases 2, 3, and 5). In case 1, the treatment of the primary cancer was not indicated because of worsening of the PS after the onset of Trousseau syndrome. Therefore, the plan was to implement rehabilitation therapy first and then consider chemotherapy depending on the degree of recovery of physical function.

## 4. Discussion/conclusion

### 4.1. FIM and mRS in patients with good outcome

In this series, none of the 2 patients with a good outcome (mRS score 0 to 2) at the most recent assessment showed severe motor impairment (such as hemiplegia) at the onset. Moreover, in these 2 patients, FIM at 1 month after admission tended to be particularly high in motor items, compared with patients with poor outcomes. Finally, patients with favorable prognoses were treated aggressively for the primary cancer.

Patients with Trousseau syndrome, like ischemic stroke patients in general,^[14]^ present with neurological symptoms such as hemiplegia, dysarthria/aphasia, ataxia, and seizures.^[5]^ However, neurological symptoms at onset tend to be severe^[15,16]^ and mRS score at 30 days after onset also tends to be worse than that of general ischemic stroke.^[16]^ Therefore, the continuation of chemotherapy after ischemic stroke tends to be reconsidered due to concerns about physical fitness and the risk of recurrent stroke due to continuation of treatment,^[3]^ and rehabilitation therapy may be administered first as a treatment strategy.^[12]^ In this setting, the risk of progression of primary cancer during rehabilitation therapy poses a dilemma, since treatment for the primary cancer is not started until improvement of physical function. However, in a study, 51% of patients had good physical function (mRS 0–2) after 3 months if there was no early deterioration of neurological symptoms after cancer-associated ischemic stroke.^[17]^ In other words, recovery can be expected in some cases. Perhaps for this reason, there is a lack of consensus on the rehabilitation strategy for Trousseau syndrome patients with neurological symptoms. Thus, there are no standards as to whether rehabilitation treatment should be provided to these patients in the same way as for ischemic stroke patients in general, or whether palliative care should be introduced. Moreover, since the primary cancer can progress during rehabilitation therapy, determining the optimal duration of rehabilitation therapy is an issue, if it is to be implemented at all.

The FIM and mRS scores of patients with good outcomes in this series suggest that the prognosis of patients with Trousseau syndrome may be favorable if, in addition to the acute treatment period (approximately 1 month), they achieve at least independent motor function 1 month after the start of intensive rehabilitation (i.e., 2 months after onset). In contrast, even if some improvement in motor function can be expected with continued rehabilitation treatment, as in case 1, patients with lack of independent motor function within 2 months of onset or those in whom motor function is not expected to improve within 2 months at the time of onset should be considered for palliative care.

### 4.2. Trousseau syndrome and primary cancer

In this study, clear cell carcinoma, which is associated with vascular embolism from early stage,^[18]^ and adenocarcinoma^[4–7]^ accounted for more than half of the primary lesions, as previously reported. In contrast, approximately half of the primary cancers were gynecological cancers. This may be attributable to the fact that >30 days had elapsed between the onset and hospitalization in addition to the poor life expectancy of patients with Trousseau syndrome. In other words, differences in life expectancy and treatment strategies could be attributable to primary lesions. In particular, patients with lung, pancreatic, and colorectal cancers, who are at high risk of developing stroke, tend to be diagnosed at an advanced stage^[19]^; therefore, it is assumed that Trousseau syndrome patients with pancreatic or lung cancer as primary lesions had either died before transfer or had no indications for rehabilitation therapy. PS also influences the treatment strategy for the primary tumor. The American Society of Clinical Oncology guidelines advise against aggressive chemotherapy for patients with solid tumors that have not benefited from prior therapy or have a PS >3.^[20]^ As shown in this study, patients with Trousseau syndrome tended to have significantly worse PS after the onset of stroke. This background might have led to a palliative treatment strategy in many cases. In contrast, cases with functional limitations due to diseases other than cancer and cases with a high likelihood of chemotherapy effectiveness even with poor PS are enumerated as exceptions to the ASOC guidelines recommendations against administering chemotherapy in patients with solid cancers.^[20]^ In fact, 3 cases of gynecological cancer were aggressively treated with tumor resection after stroke onset for staging and tumor reduction. This reflects the idea that tumor-reduction surgery for advanced cases of ovarian and uterine cancer improves survival,^[21,22]^ that it is difficult to determine the exact stage of the disease by preoperative diagnosis alone, and that surgical staging is important to determine the treatment plan. Furthermore, it would be related to the fact that ovarian clear cell adenocarcinoma tends to be complicated by cerebral infarction from the early stage of the disease.^[12]^ Therefore, the indication for intensive rehabilitation should be discussed in the future, as the treatment of patients with Trousseau syndrome may change with advances in treatment methods for the primary cancer.

This was a single-center retrospective case series, which is the main limitation of the study. Secondly, because we subjected cases after treatment of acute phase, the number of cases was limited to those who had been treated for about 1 month after stroke onset. Therefore, there were few cases of rapidly progressive malignancies like lung cancer and pancreatic cancer, and there was a bias in the primary cancer. In addition, the timing for determining the effectiveness of rehabilitation was set at 1 month after the start of intensive rehabilitation (approximately 2 months after stroke onset). Future studies should determine whether this setting was appropriate.

Among patients with Trousseau syndrome, if physical function is expected to improve after about 1 month of intensive rehabilitation and if the primary cancer can be treated aggressively, a good prognosis can be expected, and therefore, intensive rehabilitation therapy might be indicated. If independent performance of motor function is not expected within 2 months of stroke onset, transition to palliative care should be considered.

## Acknowledgments

The authors thank the staff of the Showa University Fujigaoka Rehabilitation Hospital and Kayo Takeshima.

## Author contributions

**Data curation:** Shinichi Takeshima.

**Investigation:** Shinichi Takeshima.

**Project administration:** Shinichi Takeshima.

**Supervision:** Nobuyuki Kawate.

**Validation:** Shinichi Takeshima.

**Writing – original draft:** Shinichi Takeshima.
